# NK Cell Activity and CD57^+^/NKG2C^high^ Phenotype Are Increased in Men Who Have Sex With Men at High Risk for HIV

**DOI:** 10.3389/fimmu.2020.537044

**Published:** 2020-09-11

**Authors:** Lizdany Flórez-Álvarez, Yurany Blanquiceth, Katherin Ramírez, Ana Claudia Ossa-Giraldo, Paula A. Velilla, Juan C. Hernandez, Wildeman Zapata

**Affiliations:** ^1^Grupo Inmunovirología, Facultad de Medicina, Universidad de Antioquia, Medellín, Colombia; ^2^Grupo Infettare, Facultad de Medicina, Universidad Cooperativa de Colombia, Medellín, Colombia

**Keywords:** CD57^+^/NKG2C^high^ NK cell, HIV, natural resistance, men who have sex with men, Highly exposed seronegative, cytotoxicity

## Abstract

**Introduction:**

The HIV-exposed seronegative (HESN) status is for individuals who remain seronegative despite repeated exposure to HIV. One of the main cohorts within this group is men who have sex with men (MSM). Studies of this cohort have revealed different immunological and genetic mechanisms that can explain the phenomenon of natural HIV resistance. NK cells’ higher effector capacity is related to natural resistance to HIV. Besides, a new population of NK cells with adaptive features was described recently. These cells are increased in some HESN cohorts and appear to be involved in better control of viral replication in primarily HIV-infected subjects. The present study evaluated the role of NK cells in the natural resistance to HIV-1 infection in MSM.

**Methodology:**

Phenotypic and functional features were evaluated in NK cells from two groups of MSM, at different risks of HIV infection, according to the number of sexual partners. The production of IFN-γ and β-chemokines was included in the analysis, as well as the cytotoxic capacity and adaptive NK cell frequency. Genetic features, such as HLA and KIR allele frequencies, were also explored.

**Results:**

High-risk MSM exhibit an increased frequency of fully mature and CD57^+^/NKG2C^high^ NK cells. These individuals also show higher cytotoxic capacity and IFN-γ production in response to K562 stimuli. NK cells with a CD107a^+^/IFN-γ^+^ functional profile were found more frequently and displayed higher IFN-γ production capacity among high-risk MSM than among low-risk MSM. The protective allele *HLA-B^∗^18* was only present in the high-risk MSM group as well as *HLA-B^∗^ 39*. The protective phenotype *KIR3DL1/S1-HLA-B^∗^Bw4*, in a homozygous state, was particularly abundant in the high-risk population. Notably, some of these functional features were related to higher frequencies of mature and CD57^+^/NKG2C^high^ NK cells, which, in turn, were associated with a higher number of sexual partners.

**Conclusion:**

The changes observed in the NK cell compartment can be driven by the magnitude of sexual exposure and immunological challenges of high-risk individuals, which could influence their resistance/susceptibility to HIV infection.

## Introduction

Human inmunodeficienty virus (HIV) researchers worldwide have focused on HIV-exposed seronegative (HESN) individuals, given valuable information about natural resistance to HIV that can be obtained from studies involving this population (1). These studies have revealed different immunogenetic mechanisms that can explain the phenomenon of natural HIV resistance. The most widely described of these is the *CCR5*Δ*32* mutation, which confers resistance to infection with R5 strains of HIV (2, 3). This knowledge has led to the development of antiretroviral drugs that act to block this co-receptor, emphasizing the importance of research on mechanisms of natural resistance to HIV in order to formulate new therapeutic strategies and vaccines.

Men who have sex with men (MSM) represent an interesting cohort for studying natural resistance mechanisms, based on their social and biological characteristics, that make them a group at high risk for HIV infection. This cohort represents nearly 69% of HIV-positive men around the world (4). Natural resistance mechanisms described in other HESN cohorts, such as serodiscordant couples and commercial sex workers, have also been found in MSM. However, many other mechanisms remain to be studied, including increased effector capacity of NK cells, which represents an important natural resistance mechanism (5, 6).

NK cells may contribute to HIV infection control in several ways. These are essential to the induction of adaptive immune responses and can eliminate infected cells through cytotoxic mechanisms (7) and the production of β-chemokines, which prevent the infection of new cells by blocking viral co-receptors (5, 8–11). In 2003, Scott-Algara et al. reported, for the first time, increased effector capacity of NK cells in intravenous drug users (IDUs) who remained uninfected after several years of practices associated with a high risk of exposure to HIV. NK cells from HESN IDUs showed a higher cytotoxic capacity than NK cells from healthy controls and other IDUs who seroconverted during the study, showing the importance of NK cell effector capacity for natural resistance to HIV (5).

In 2006, NK cells with memory characteristics were described in murine models (12). Later, in 2015, Reeves et al. reported these cells could eliminate dendritic cells pulsed with vaccine proteins from simian immunodeficiency virus in vaccinated rhesus macaques in a specific way (13). A population of CD57^+^/NKG2C^+^ NK cells has been found at higher frequencies in a cohort of HESN individuals than in healthy donors and HIV-infected individuals (14). Studies carried out in primarily HIV-infected individuals showed that a higher frequency of NKG2C^+^ NK cells correlates with a lower viral set point establishment and better immunological parameters (i.e., lower plasma levels of IL-6, and lower PD-1 expression on mDCs). This suggests that CD57^+^/NKG2C^+^ NK cells can contribute to HIV replication control (15), implying a resistant phenotype.

## Materials and Methods

### Study Population

A cross-sectional study involving a cohort of 42 MSM recruited from Medellín, Colombia is presented. The MSM were divided into two groups according to the frequency of sexual partners in the 3 months before enrolling in the study: (i) MSM at high risk of HIV infection: those with more than 15 different sexual partners in the last 3 months with unprotected sexual intercourse (high-risk MSM) and (ii) MSM at lower risk of HIV infection: those with four or fewer than four different sexual partners in the last 3 months with unprotected sexual intercourse (low-risk MSM); all individuals included reported having receptive anal sexual encounters. MSM younger than 18 years of age, positive for HIV 1/2 rapid test (SD BIOLINE, Abbott), positive for HIV-1 proviral DNA by PCR, or homozygous for *CCR5* Δ32 mutation were excluded.

Signed informed consent and a questionnaire on risky behaviors were obtained from each individual after the study protocol was explained to them. Later, 50 mL of peripheral blood was taken with a disposable syringe. This study was performed following the Helsinki Declaration (1975, revised in 2000) and approved by the bioethics board of the Universidad de Antioquia.

### Frequency and Phenotype of NK Cells

Aliquots of whole blood were stained for 25 min in the dark using the following monoclonal antibodies: CD45 (HI30, APC-eFluor780), CD56 (CMSSB, PE-Cy5), CD3 (UCHT1, Alexa Fluor 700), and CD57 (TB01, FITC), obtained from Thermo Scientific (Wilmington, DE, United States); NKG2C (134591, PE; R&D Systems, MN, United States); and CD16 (3G8, V450; BD Biosciences, San Jose, CA, United States). After staining, the samples were treated with red blood cell lysing solution (BD Biosciences) following the manufacturer’s instructions. Finally, cells were washed twice with phosphate-buffered saline (PBS) (Lonza, Rockland, ME, United States) and suspended in 2% paraformaldehyde. Cells were acquired using LS Fortessa (BD Biosciences, San Jose, CA, United States) and data were analyzed using FlowJo version 10.5.3 (FlowJo, LLC, Ashland, Oregon, United States).

### Natural Cytotoxicity Assays

Peripheral blood mononuclear cells (PBMCs) were isolated through a density gradient with Ficoll-Histopaque (Sigma-Aldrich, St. Louis, MO, United States) by centrifugation at 400 *g* for 30 min. They were then washed three times with PBS to eliminate platelets and debris. Next, the cells were counted and frozen until used.

PBMCs were thawed and left in culture with RPMI (Sigma-Aldrich, St. Louis, MO, United States) supplemented with 10% fetal bovine serum (FBS) (Gibco, Grand Island, NY, United States) for 24 h before each experiment. K562 cells were used as targets because there was no expression of classical HLA-I molecules. Before culturing, 1 × 10^6^ K562 cells were stained with 0.1 mM eFluor670 (Thermo Scientific, Wilmington, DE, United States) in PBS for 10 min at 37°C to identify them as target cells in the flow cytometry analysis. Then, PBMCs were co-cultured with K562 cells in round-bottomed tubes at a 10:1 ratio in 300 μL of RPMI with 10% FBS for 4 h at 37°C and 5% CO_2_.

After incubation, the cells were stained with propidium iodide (PI) and DIOC-6 (both from Thermo Scientific, Wilmington, DE, United States) for 15 min in the dark. PI and DIOC-6 were used to evaluate the integrity of the cell and mitochondrial membranes, respectively. K562 cells were cultured in the absence of PBMCs as a spontaneous death control. This control was carried out for every assay. Spontaneous death control had to be lower than 15% for the experiment to be valid. The cytotoxicity percentage was adjusted based on spontaneous death control.

### NK Cell Activation Assays

Target K562 cells were cultured in RPMI supplemented with 10% FBS at a density of 1 × 10^6^/mL until the co-culture. Fresh PBMCs (2 × 10^6^ cells/mL) were stimulated with 25 ng/mL IL-15 (Thermo Scientific, Wilmington, DE, United States) in six-well plates with 2 mL of RPMI supplemented with 10% FBS overnight to prime NK cells for IFN-γ production. Then, PBMCs were cultured in round-bottomed tubes at a 10:1 ratio with K562 (1 × 10^6^ PBMC:1 × 10^5^ K562) in 300 μL of RPMI with 10% FBS, 6 μg/mL brefeldin A (Thermo Scientific, Wilmington, DE, United States), 2 mM monensin (Thermo Scientific, Wilmington, DE, United States), and 1 μL of anti-CD107a (BD Biosciences, San Jose, CA, United States) for 5 h at 37°C and 5% CO_2_. A replica of every tube with no brefeldin A and monensin was included for Cytometric Bead Array (CBA) analysis.

Before staining, the cells were incubated with 100 μL of IgG (20 μg/mL) to block Fc receptors. Then, the cells were stained with monoclonal antibodies (Mabs) against CD45, CD56, and CD16 for 20 min in the dark. The cells were treated with Foxp3/Transcription Factor Staining Buffer Set (Thermo Scientific, Wilmington, DE, United States), following the manufacturer’s instructions to permeabilize cells. Subsequently, cells were stained with anti-CD3, -IFN-γ (4S.B3, BV711; BioLegend, San Diego, CA, United States), -Granzyme B (BG11, FITC; BD Biosciences), and -MIP-1β (PE; BD Biosciences) for 25 min in the dark. Finally, cells were suspended in 2% paraformaldehyde and acquired using LS Fortessa. Data were analyzed using FlowJo version 10.5.3.

### Quantification of Effector Molecules by CBA

Supernatants of NK cell activation assays were collected and stored at −80°C until used. Supernatants were thawed at 4°C, just before the CBA assay. CBA flex set for MIP-1α, RANTES, TNF-α, and IFN-γ (BD Biosciences) was used and performed following the manufacturer’s instructions. Bead complexes were acquired using LS Fortessa. Data were analyzed using FlowJo version 10.5.3.

### Quantification of mRNA by Real-Time RT-PCR

Total RNA was purified from PBMCs using the Direct-zol RNA Miniprep kit (Zymo Research, Orange, CA, United States), following the manufacturer’s instructions. The RNA was reverse-transcribed to cDNA using a high-capacity cDNA reverse transcription kit (Thermo Scientific, Wilmington, DE, United States). PCR reactions were performed using the Maxima SYBR Green qPCR master mix kit (Fermentas, France). Real-time RT-PCR was performed in a QuantStudio 5 Real-Time PCR System (Thermo Scientific, Wilmington, DE, United States). The data are expressed as relative units, normalized against the constitutive gene PGK (phosphoglycerate kinase), using formula 1.8^−[ΔCt]^, where 1.8 corresponds to the mean PCR efficiency of 80%. The primer sequences and PCR conditions are described in Supplementary Table S1.

### HLA and KIR Genotyping

HLA and KIR genotyping were performed in collaboration with the Hospital San Vicente Fundación, Colombia. HLA genotyping was performed using the DNA hybridization assay (LIFECODES HLA-SSO Typing) with SSO probes attached to multicolored beads; the bead complexes were detected with a Luminex 100 flow cytometer. KIR alleles were genotyped using RSSOKIR (One-Lambda, Canoga Park, CA, United States), a multiplex polymerase chain reaction sequence-specific primer reaction.

### Determination of Anti-HCMV IgG Titers

Titers of IgG antibodies specific for HCMV were determined using a Human Anti-Cytomegalovirus IgG ELISA Kit (CMV) (Abcam, Cambridge, MA, United States) in serum samples of all participants, following the manufacturer’s instructions.

### Statistical Analysis

To compare data from high-risk MSM vs. low-risk MSM, Mann–Whitney *U* or Student’s *t*-test was performed, depending on the bivariate normality assumption according to the Shapiro–Wilk normality test. Correlation analyses were based on the calculation of Spearman’s correlation coefficient. A *p*-value < 0.05 was considered statistically significant. The statistical tests were performed using GraphPad Prism Software version 7.02.

## Results

### MSM Socio-Demographic Data

Forty-two MSM who met the inclusion criteria were enrolled in this study. Their socio-demographic data are summarized in Table 1. The median number of lifetime sexual partners among the high-risk MSM was 1200, compared with 29 for the low-risk MSM group (*p* = 0.0001). Therefore, the high-risk group had higher sexual exposure, not only in the 3 months prior to the study, per the inclusion criteria, but also during their entire active sexual lives. In the high-risk MSM group, there were more frequent findings of a history of HIV-positive sexual partners, sexually transmitted infections (STI), and heterozygosity for *CCR5* Δ32 mutation compared with the low-risk MSM group. The rate of condom use was near 50% in both groups. The ages upon starting to have sexual intercourse were similar between the two groups; however, the high-risk MSM were older and had a longer duration of high-risk sexual activity than the low-risk MSM.

**TABLE 1:** Socio-demographic data of the study participants.

	*High-risk MSM*	*Low-risk MSM*	*p*
*n*	16	26	
*Age, median (Q1–Q3)*	29.5 (26.2–36.7)	25.5 (20.7–29.7)	0.0127^a^
*Sexual partners in the last 3 months, median (Q1–Q3)*	27 (21–36)	2 (1–4)	<0.0001^a^
*Sexual partners in a lifetime, median (Q1–Q3)*	1200 (571–2954)	29 (11–100)	<0.0001^a^
*% of unprotected sex in the last 3 months, median (Q1–Q3)*	46.5 (12.7–74.7)	48 (0–91)	0.984^a^
*Frequency of STIs*	87.5%	38.4%	0.003^b^
*Frequency of HIV-positive partners*	62.5%	42.3%	0.340^b^
*Frequency of CCR5*Δ*32 heterozygosity*	18.7%	3.85%	0.146^b^
*Age of onset of sexual intercourse, median (Q1–Q3)*	15 (13.2–17.7)	17 (14–20.2)	0.172^a^
*Duration of sexual activity (years), median (Q1–Q3)*	13.5 (11.2–20.5)	9 (5–12.2)	0.001^a^

*a Mann–Whitney test. b Fisher’s exact test. STI, Sexually transmitted infection; CCR5, C-C chemokine receptor type 5.*

**FIGURE 1:**
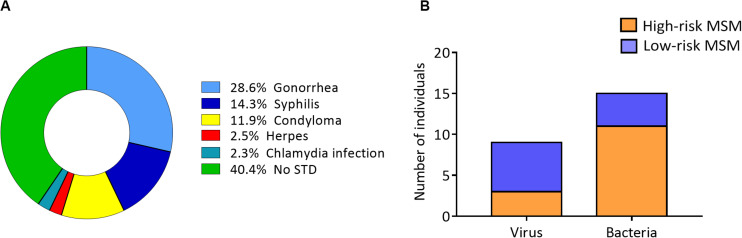
Lower frequency of viral STIs in high-risk MSM. **(A)** STI distribution among all study participants (STI history was self-reported by each individual in the questionnaire). **(B)** STI distribution according to the causative agent (viral or bacterial) in the two groups.

The most common STIs were gonorrhea and syphilis in the entire study population (Figure 1A). When STI frequency was evaluated, based on the causative agent, the results showed that STIs with viral origins, such as herpes and condyloma, were more frequent in low-risk MSM (Figure 1B).

### High-Risk MSM Exhibits a Mature Phenotype in the NK Cell Compartment Compared With Low-Risk MSM

**FIGURE 2:**
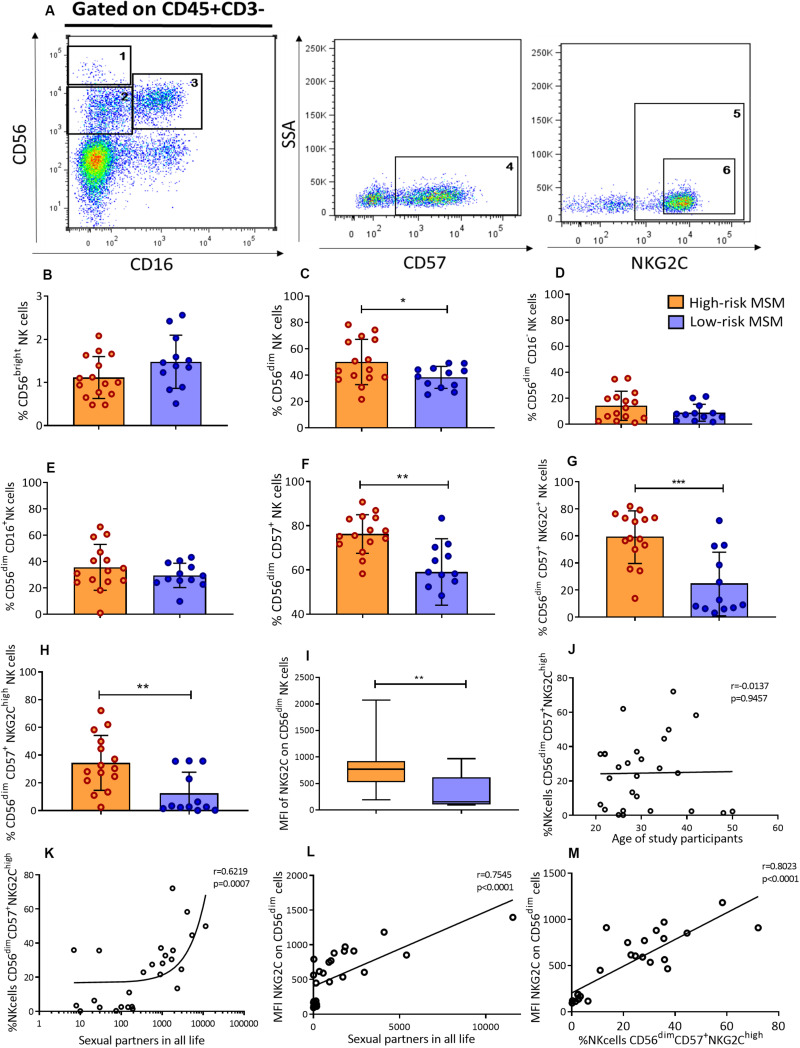
High-risk MSM have a higher frequency of CD56^dim^ cells and adaptive CD57^+^/NKG2C^high^ NK cells. **(A)** Representative gating strategy identifying NK cell subpopulations. NK cells were selected as CD45^+^CD3^–^ cells from peripheral blood; subsequently, according to CD56 and CD16 expression, three NK cell subpopulations were defined: (1) CD56^bright^, (2) CD56^dim^CD16^–^, and (3) CD56^dim^CD16^+^. In the CD56^dim^ subpopulation (2 + 3), the cells expressing CD57 marker were defined as terminally differentiated NK cells: (4) CD56^dim^CD57^+^. In the last subpopulation, two gating were performed: (5) CD56^dim^CD57^+^NKG2C^+^ and (6) CD56^dim^CD57^+^NKG2C^high^. **(B–G)** Frequencies of NK cell subpopulations in peripheral blood. **(H)** Frequency of adaptive CD57^+^/NKG2C^high^ NK cells. **(I)** The MFI of NKG2C expression on CD56^dim^ NK cells. **(J,K)** Correlations of CD57^+^/NKG2C^high^ NK cell frequency with age and the number of sexual partners throughout the life span, respectively. **(L)** Correlation of the MFI of NKG2C expression on CD56^dim^ NK cells and the number of lifetime sexual partners. **(M)** Correlation of the MFI of NKG2C expression on CD56^dim^ NK cells and the frequency of CD57^+^/NKG2C^high^ NK cells. Statistical evaluations were performed using Mann–Whitney *U*, unpaired *t*-test or Spearman’s correlation test. *p < 0.05, **p < 0.01, and ***p < 0.001.

The frequency and phenotype of NK cells in the peripheral blood of MSM were analyzed by flow cytometry. The two main NK cell subpopulations, CD56^bright^ and CD56^dim^, as well as terminally differentiated NK cells, identified by the expression of the maturation marker CD57, were analyzed. CD57^+^/NKG2C^high^ NK cells, which are terminally differentiated NK cells with high expression of activating receptor NKG2C, were also included in the analysis (Figure 2A).

Although there were no significant differences in the percentage of CD56^bright^ NK cells (1.1 ± 0.5 vs. 1.5 ± 0.6, *p* = 0.09) (Figure 2B) between the two groups, the rate of total CD56^dim^ NK cells was higher in high-risk than low-risk MSM (50 ± 17.1 vs. 38.4 ± 8.3, *p* = 0.041) (Figure 2C). Although no significant differences were observed in the frequency of CD56^dim^ subpopulations, CD56^dim^CD16^–^ and CD56^dim^CD16^+^ (Figures 2D,E), the frequency of terminally differentiated NK cells (CD56^dim^/CD57^+^) was also higher in high-risk MSM (76.2 ± 8.7 vs. 59.0 ± 15.0, *p* = 0.001) as well as CD57^+^/NKG2C^+^ NK cells (Figures 2F,G).

NK cell subpopulations may correspond to different maturation stages. CD56^bright^ is the most immature stage and CD56^dim^ is the most mature stage; the latter of these is the cellular compartment, where most of the terminally differentiated NK cells are located. The CD57 maturation marker is positively regulated during the maturation process, and it allows the identification of those maturation stages. Higher percentages of DC56^dim^ and CD57^+^ NK cells indicate a mature phenotype in high-risk compared with low risk-MSM, this population also have a higher density of CD57 expression were NK cell subpopulation were compared between both groups (Supplementary Figure S1).

### CD57^+^/NKG2C^high^ NK Cells Are More Frequent in High-Risk MSM Than in Low-Risk MSM

The cumulative evidence indicates that there are NK cell subpopulations with certain adaptive characteristics. CD57^+^/NKG2C^high^ NK cells are terminally differentiated NK cells that, under certain stimuli, undergo some phenotypic and functional changes that are frequently associated with HCMV infection. These changes include the acquisition of adaptive features and the upregulation of NKG2C expression, which represents a key activation receptor of CD57^+^/NKG2C^high^ NK cells.

The frequency of CD57^+^/NKG2C^high^ NK cells, as well as the MFI of NKG2C, was evaluated in both groups. The results showed that high-risk MSM had not only a higher frequency of these cells than low-risk MSM (34.3 ± 19.7 vs. 12.4 ± 15.2, *p* = 0.006) (Figure 2H), but also a higher density of NKG2C expression on the CD56^dim^ NK cell compartment (837.4 ± 455.5 vs. 334.2 ± 312.6, *p* = 0.0026) (Figure 2I).

Higher levels of anti-HCMV IgG are related to higher numbers of CD57^+^/NKG2C^high^ NK cells, as shown previously. For that reason, anti-HCMV IgG levels were evaluated to explain the higher frequency of CD57^+^/NKG2C^high^ NK cells found in high-risk MSM. High-risk MSM exhibited titers of anti-HCMV IgG ranging from 176.2 to 1845 UI/mL, while those in low-risk MSM ranged from 79 to 814.5 UI/mL, with no significative difference between them (*p* = 0.217).

The frequency of CD57^+^/NKG2C^high^ NK cells is related to the aging process along with the exposure to HCMV. As shown in the socio-demographic data, the high-risk MSM were older than the low-risk ones; therefore, the correlation between the frequency of CD57^+^/NKG2C^high^ NK cells and age was evaluated in both MSM groups. There was no correlation between these variables (*r* = 0.0137, *p* = 0.9457) (Figure 2J). However, there was a strong positive correlation between the frequency of CD57^+^/NKG2C^high^ NK cells and the number of lifetime sexual partners (*r* = 0.6219, *p* = 0.0007) (Figure 2K), but not with sexual partners in the previous 3 months (Supplementary Figure S2). These data suggest that the magnitude of sexual exposure, measured as the number of sexual partners, could stay contributing to CD57^+^/NKG2C^high^ NK cell expansion rather than aging or HCMV infection.

A strong correlation was also found between the density of NKG2C expression on CD56^dim^ NK cells (*r* = 0.7545, *p* < 0.0001) and the number of lifetime sexual partners (Figure 2L), as well as with the frequency of CD57^+^/NKG2C^high^ NK cells (*r* = 0.8023, *p* < 0.0001) (Figure 2M). This confirmed the strong nexus between sexual exposure and the frequency of CD57^+^/NKG2C^high^ NK cells found in the peripheral blood of MSM individuals.

### Cytotoxic Activity of NK Cells Is Higher in High-Risk MSM Than in Low-Risk MSM

**FIGURE 3:**
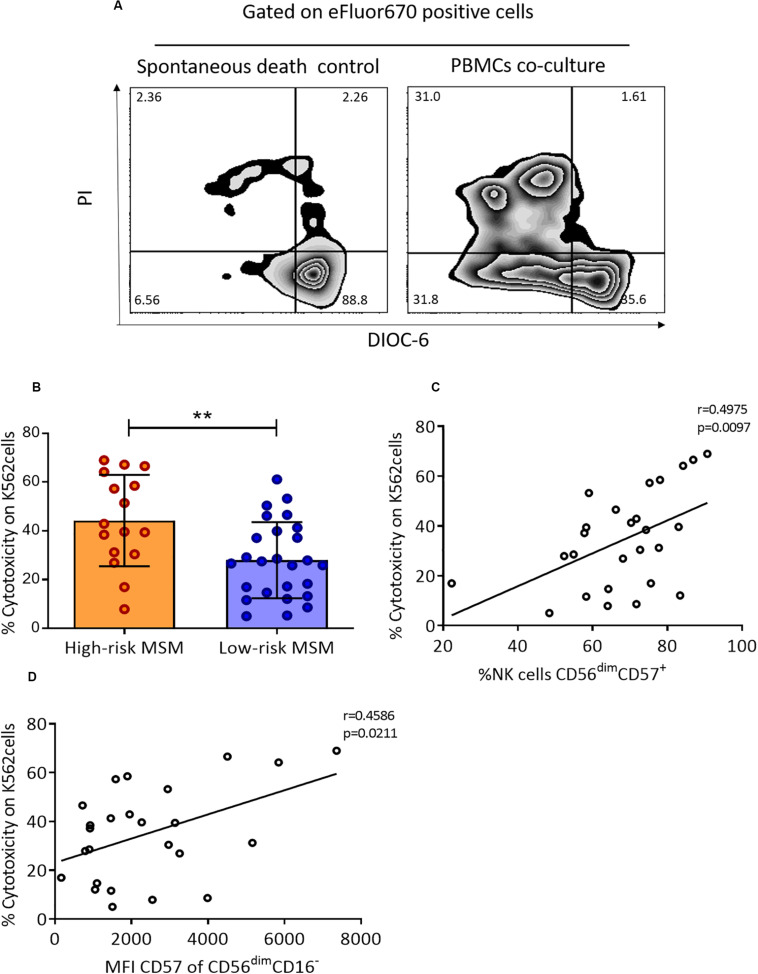
PBMCs from high-risk MSM exhibit higher cytotoxic activity against K562 cells. **(A)** Representative gate of target cells (K562) selected by eFluor670 expression after 4 h of culture. On the left, the apoptosis control with target cells alone (in the absence of effector cells); on the right, target cells after 4 h of co-culture with PBMCs. **(B)** The cytotoxic capacity over K562 cells. The cytotoxicity rate is expressed as the cell death percentage, corrected using an apoptosis control (live target cells are DIOC-6^+^/PI^–^). The results are shown as mean ± SD, n: 16;26. Statistical evaluations were performed with unpaired *t*-test. **(C)** Correlation of cytotoxicity with terminally differentiated NK cells. Spearman’s correlation test. **(D)** Correlation of cytotoxicity with the MFI of CD57 in the CD56^dim^CD16^–^ subpopulation. Spearman’s correlation test. **p < 0.01.

A higher cytotoxic capacity of NK cells has been reported in several cohorts for the study of HESN individuals related to protection against HIV infection. In this study, the cytotoxic capacity in both groups of MSM was evaluated by co-cultures of PBMCs with the K562 cell line. A representative scheme of the cytotoxicity assessment is shown in Figure 3A.

High-risk MSM showed a higher cytotoxic capacity than low-risk MSM against the tumoral cell line K562 (44.2 ± 18.7 vs. 27.9 ± 15.5, *p* = 0.002) (Figure 3B). The cytotoxic capacity of NK cells and CD8^+^ T cells was previously shown to be related to the expression of CD57; for this reason, the correlation between the frequency of cells expressing this marker and the cytotoxic capacity was evaluated. There was a positive correlation between the cytotoxic capacity and the frequency of total CD56^dim^CD57^+^ NK cells (*r* = 0.497, *p* = 0.009) (Figure 3C). Finally, a correlation between the MFI of CD57 expression and cytotoxicity was found in CD56^dim^CD16^–^ NK cells (*r* = 0.458, *p* = 0.021) (Figure 3D), but not in any other NK cell subpopulation.

### High-Risk MSM Exhibit a Higher Frequency of IFN-γ-Positive NK Cells and Higher Levels of MIP-1α Production After K562 Co-culture

**FIGURE 4:**
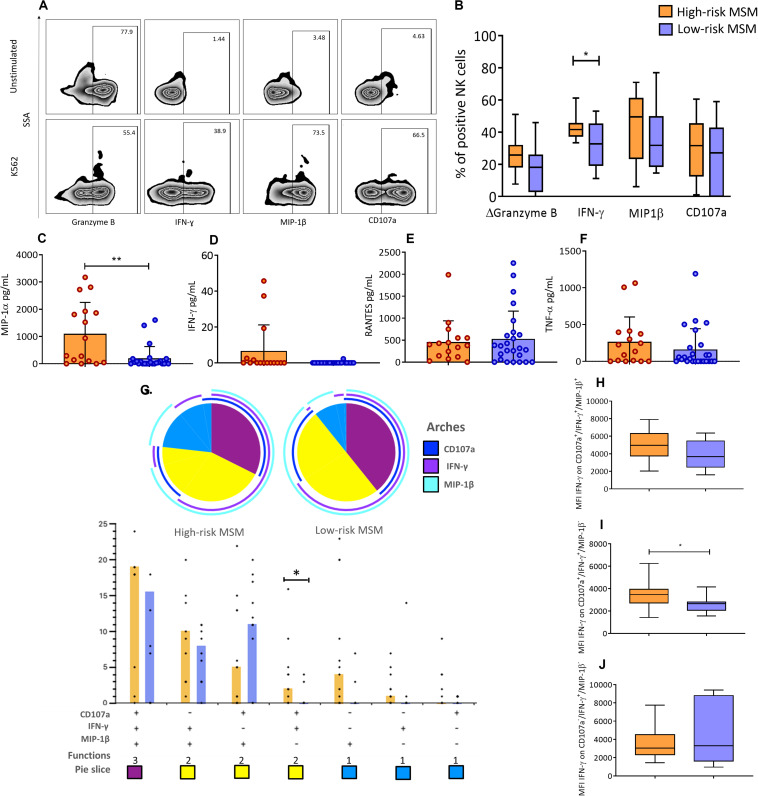
NK cells in high-risk MSM show different functional profiles compared to low-risk MSM. **(A)** Expression of activation markers in total NK cells before the stimulus and after 4 h of K562 co-culture. **(B)** Frequency of NK cells positive for effector molecules after 4 h of co-culture. Granzyme B is shown as ΔGranzyme B (% Granzyme B-positive cells in unstimulated condition – % Granzyme B-positive cells after K562 stimuli); the subtraction represents the percentage of NK cells that degranulate Granzyme B in response to the stimulus. The line inside the box indicates the mean, and whiskers indicate min. to max. value. **(C–F)** The concentration of effector molecules in the supernatant after 4 h of PBMC and K562 co-culture. **(G)** Functional profile analysis of NK cells after co-culture with the K562 cell line. The results are presented as mean. n: 15;10. Statistical evaluations were performed using unpaired *t*-test. *p < 0.05. In the permutation analysis carried out in the SPICE platform only data higher than 0.1% were included. **(H–J)** The MFI of IFN-γ in CD107a^+^/IFN-γ^+^/MIP-1β^+^, CD107a^+^/IFN-γ^+^/MIP-1β^–^, and CD107a^–^/IFN-γ^+^/MIP-1β^–^ NK cells, respectively. The line inside the box indicates the mean, and whiskers indicate min. to max. value. n: 15;10. Statistical evaluations were performed using unpaired *t*-test or Mann–Whitney *U* test. *p < 0.05 and **p < 0.01.

Granzyme B, CD107a, MIP-1β, and IFN-γ were evaluated as activation markers in NK cells after co-culture with K562 cells, by flow cytometry. A representative scheme of the activation assessment is shown in Figure 4A. High-risk MSM had a higher frequency (42.5 ± 6.8 vs. 32.6 ± 14.6, *p* = 0.039) of cells positive for IFN-γ in response to K562 cells (Figure 4B). No statistically significant differences were observed in the production of the other molecules (Figure 4B).

The expression of MIP-1α, TNF-α, RANTES, and IFN-γ was also evaluated by CBA in the supernatants of the K562-PBMC co-culture. High-risk MSM showed higher MIP-1α production than low-risk MSM (1102 ± 1149 vs. 199 ± 432, *p* = 0.002) (Figure 4C). IFN-γ production was detected in 18.7% of high-risk MSM supernatants but was not detected in any low-risk MSM supernatants (Figure 4D). There were no differences in the production of TNF-α and RANTES between the groups (Figures 4E,F).

### High-Risk MSM Showed a Distinct Functional Profile of NK Cells Compared With Low-Risk MSM

The functional profile of NK cells was evaluated in response to K562 cell stimulus. The measured molecules included CD107a, as a degranulation marker, MIP-1β, and IFN-γ.

The functional profile analytical results showed that monofunctional responses were more frequent among high-risk MSM. Analyses of NK cells with two functions revealed that there is a different NK cell population between the two groups, which was characterized by a CD107a^+^/IFN-γ^+^/MIP-1β^–^ profile. These cells were more frequent in high-risk MSM than in low-risk MSM (*p* = 0.043) (Figure 4G). The frequencies of NK cells positive for the three markers were similar between the two groups.

Next, the MFI of IFN-γ in NK cells with different functional profiles was determined. The MFI of IFN-γ in NK cells with a CD107a^+^/IFN-γ^+^/MIP-1β^–^ profile was higher in high-risk MSM than in low-risk MSM (3499 ± 1363 vs. 2546 ± 737, *p* = 0.035) (Figure 4I). Differences in the MFI of IFN-γ were not observed in NK cells with other functional profiles between the groups (Figures 4H,J). These results showed that high-risk MSM exhibit not only a higher frequency of NK cells but also major IFN-γ production by these cells with this functional profile, which could explain their better functional activity.

### Basal mRNA of IFN-γ Is Higher in High-Risk MSM Than in Low-Risk MSM

The relative transcriptional expression of molecules associated with the effector function of NK cells was evaluated. Cytotoxic molecules (granzyme B and perforin), β-chemokines (MIP-1α, MIP-1β, and RANTES), IFN-γ, and TNF-α were evaluated.

**FIGURE 5:**
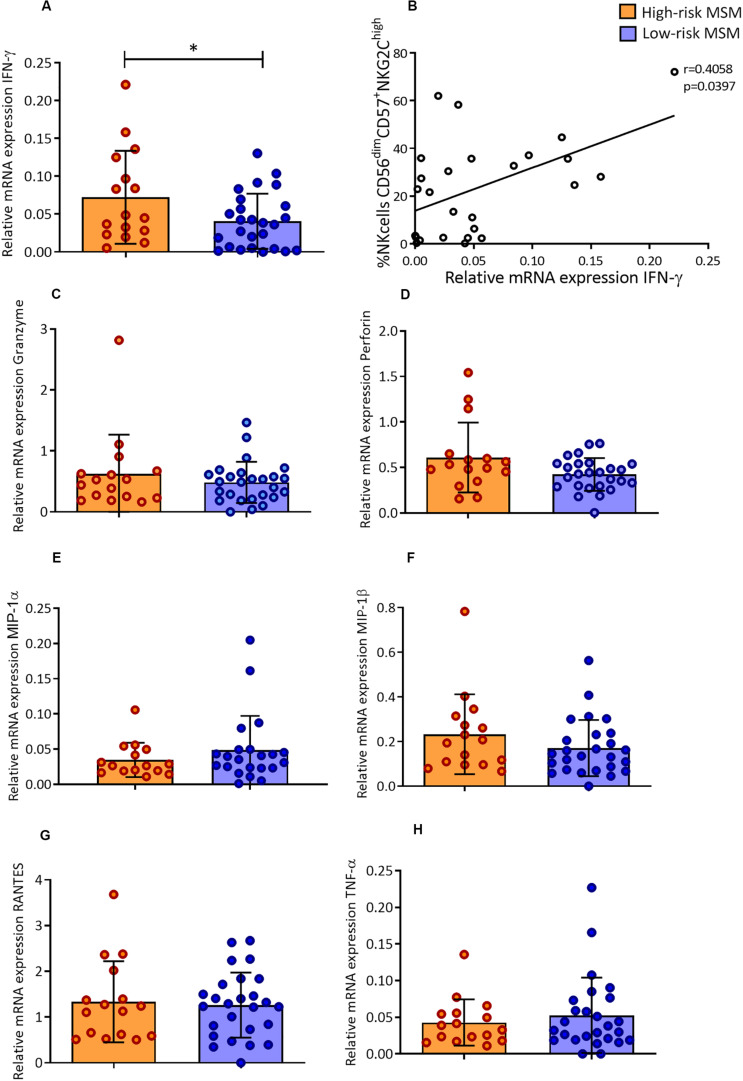
High-risk MSM have higher levels of IFN-γ mRNA in a basal state, which are correlated with the frequency of memory-like NK cells. **(A)** Relative mRNA expression of IFN-γ. n: 16;26. Statistical evaluations were performed using unpaired *t*-test. Results are shown as mean ± SD, *p < 0.05. **(B)** Correlation of relative mRNA expression of IFN-γ with the frequency of CD57^+^/NKG2C^high^ NK cells. Spearman’s correlation test. **(C–H)** Relative mRNA expression of the other molecules evaluated; the results are shown as mean ± SD. n: 16;26. Statistical evaluations were performed using unpaired *t*-test.

The mRNA levels of IFN-γ in PBMCs were higher in high-risk MSM that in low-risk MSM at the basal state (0.07 ± 0.06 and 0.04 ± 0.03, *p* = 0.040) (Figure 5A). The IFN-γ transcript levels were also correlated with the frequency of CD57^+^/NKG2C^high^ NK cells (*r* = 0.405, *p* = 0.039) (Figure 5B). No differences between groups were found in the relative expression of other molecules (Figures 5C–H).

### KIR/HLA Protective Combination Frequency

Finally, considering that combinations of certain HLA-KIR genotypes have been associated with resistance to HIV infection, the frequency of *HLA-B* and *KIR* alleles was determined in the MSM groups. The *HLA-B* allele *HLA-B^∗^18* was present at a rate of 23% in the high-risk MSM but was absent in the low-risk group, as well as *HLA-B^∗^39* (Supplementary Tables S2, S3).

The frequency of the protective combination *KIR3DL1/S1-HLA-B^∗^Bw4* in the homozygous state was determined in the study population. The protective phenotype *KIR3DL1/S1-HLA-B^∗^Bw4/Bw4* was present at a rate of 30.7% in the high-risk MSM, while this phenotype was present in only 8% of the low-risk MSM. Three of the four high-risk individuals carrying the protective phenotype presented at least one HLA Bw4 80I allele (Table 2).

**TABLE 2:** Frequency of KIR/HLA alleles associated with protection*.

	High-risk MSM *n* = 13 *% (n)*	Low-risk MSM *n* = 25 *% (n)*	*OR*	*95% CI*	*p*
KIR3DS1	23 (3)	44 (11)	0.381	0.096–1.796	ns
KIR3DL1	92.3 (12)	88 (22)	1.636	0.219–22.83	ns
KIR3DL1/S1	92.3 (12)	96 (24)	0.5	0.025–10.23	ns
HLA-B* Bw4	61.5 (8)	25 (13)	1.477	0.356–5.732	ns
HLA-B*Bw4/Bw4	30.7 (4)	8 (2)	5.111	0.953–29.05	ns
3DL1/S1-B*Bw4/-	53.8 (7)	48 (12)	1.264	0.298–4.275	ns
3DL1/S1-B*Bw4/Bw4	30.7 (4)	8 (2)	5.111	0.953–29.05	ns

* The frequency of each phenotype is shown as a percentage and OR indicating how many times more probable it is to find that condition in the high-risk MSM group; column p corresponds to Fisher’s exact test.

## Discussion

This study provides information on combinatorial mechanisms and key characteristics of the NK cells, which could correlate with natural protection against HIV infection (or even other viruses) during sexual exposure of high-risk MSM. To the best of our knowledge, this the first report of increased NK cell activity in the MSM population.

The frequency of sexual partners in our high-risk MSM group exceeds the numbers reported in previous studies of high-risk MSM, ranging from three to six different sexual partners in the past 3 months (16, 17). That frequency is considered a high-risk practice, indicating that our high-risk population has even higher, exposure than other MSM cohorts. The median number of lifetime sexual partners indicates consistent high-risk practices throughout the sexually active part of the lifespan. This point, along with the high prevalence of STIs and the fact that nearly 60% of our high-risk group reported having at least one HIV-positive sexual partner, reflects the magnitude of sexual exposure and the immunological experience of this population. One dilemma in high-risk sexual cohort studies is the selection of control subjects. Many studies include low-risk (non-MSM) individuals as controls; however, it is important to mention that including this kind of control does not allow evaluation of factors such as repeated anal intercourse, allo-exposure (due to sperm contact), and STIs, which most likely affect baseline immunological parameters (1). In this study, the low-risk MSM individuals were from the same MSM community (considered globally as a high-risk population), with less infectious pressure, and insufficient to be considered HIV-resistant, based on the previously mentioned factors.

Demographic data revealed that the frequency of *CCR5* Δ32 mutation in a heterozygous state in our high-risk population (18.7%) was similar to the frequency found in other MSM cohorts from the United States (12.9%) and Italy (20%) (18, 19), but higher than frequencies reported for the general population (2%) (20). This is related to a certain degree of protection in HESN cohorts (18, 19), but not in the general population (21). These findings suggest that the high frequency of *CCR5* Δ32 mutation in a heterozygous state can contribute to the fact that high-risk MSM remains seronegative despite long periods of high-risk sexual behaviors (22).

Evaluation of the NK cell phenotype revealed higher frequencies of mature, terminally differentiated and adaptive CD57^+^/NKG2C^high^ NK cells in high-risk MSM. This population also shows a higher expression of the maturation marker CD57 in all NK cell subpopulations (23). Moreover, it has been reported that NK cell maturation is an age-dependent process, where young people have high frequencies of CD56^bright^ cells compared with older people, who show a higher frequency of CD56^dim^ cells expressing maturation markers, such as CD57 (24, 25). However, it is not clear whether this phenomenon is explained by an intrinsic aging process or by cumulative lifetime exposure. Goodier et al. reported that in a Gambian population with a high frequency of HCMV infection, children reached the percentage of terminally differentiated NK cells (CD56^dim^/CD57^+^/NKG2C^+^) of an adult (nearly 70%) by the age of six, while Europeans barely reached these numbers in adulthood (nearly 50%) (26). A similar phenomenon has been reported in transplant recipients, where CD57^+^NKG2C^+^ NK cells are detected within 3 months in patients with reactivated HCMV infection after transplantation, while these cells can take more than 1 year to emerge in patients without infection reactivation (27). The expansion of CD57^+^NKG2C^+^ NK cells has also been reported in individuals infected with hantavirus, chikungunya, hepatitis B and C viruses (28–30), suggesting that exposure to infections, rather than aging, is a determinant for NK cell maturation rates.

Some factors explaining differences in the frequencies of mature and CD57^+^/NKG2C^high^ NK cells between high-risk and low-risk MSM were evaluated. For instance, age and IgG titers against HCMV were reported to be positively correlated with the frequency of CD57^+^/NKG2C^high^ NK cells in HESN individuals (14); however, we did not found significative differences in the HCMV IgG titers between both groups. High-risk MSM were older than low-risk MSM, although there was no correlation between CD56^dim^CD57^+^ or CD56^dim^CD57^+^NKG2C^high^ frequencies and age. Interestingly, we found a strong correlation between the number of sexual partners in the lifetime and the CD56^dim^CD57^+^NKG2C^high^ NK cells frequency and the NKG2C MFI (an important activating receptor of CD57^+^/NKG2C^high^ NK cells). These results suggest that the magnitude of exposure, measured as the number of sexual partners, may be implicated in the expansion of CD57^+^/NKG2C^high^ NK cells observed in high-risk MSM.

A mature phenotype was previously related to better cytotoxic capacity (23, 31). In our study, high-risk MSM exhibited higher cytotoxicity against the tumoral cell line K562 than low-risk MSM. In fact, the frequency of mature NK cells was positively correlated with the cytotoxic capacity, in terms of both the frequency and the density of CD57 expression.

As functional characteristics, after the K562 target cell stimulation, high-risk MSM exhibited a higher frequency of IFN-γ^+^ NK cells and higher production of MIP-1α. High rates of IFN-γ^+^ NK cells and β-chemokine production are associated with protection against HIV in other HESN cohorts, including serodiscordant couples, UDIs, and babies born to HIV-positive mothers (5, 8, 9); likewise, they are related to delayed progression to AIDS in long-term non-progressors (32).

Functional analysis revealed the presence of CD107a^+^/IFN-γ^+^/MIP-1β^–^ NK cells. This population was more common in high-risk MSM, and it exhibited a higher IFN-γ production capacity than it did in the same population in low-risk MSM. NK cells with a CD107a^+^/IFN-γ^+^ functional profile have been associated with better control of HIV infection (33, 34). In 2013, Jiang et al. reported that long-term non-progressors had higher frequencies of these cells compared with typical progressors and healthy controls (32). The higher frequency of NK cells with this functional profile has also been associated with lower viral load and a lower CD4^+^ T-cell slope (34). Our findings suggest that the high number of CD107a^+^/IFN-γ^+^ NK cells found in high-risk MSM could play a role in natural resistance against HIV.

Higher mRNA levels of IFN-γ were found in PBMCs from high-risk MSM, and positively correlated with the number of CD57^+^/NKG2C^high^ NK cells. Along with phenotypic changes, a big proportion of CD57^+^/NKG2C^high^ NK cells suffer a series of epigenetic changes, including demethylation in the *IFNG* gene, resulting in an enhanced capacity of IFN-γ production (35), which was consistently observed in our functional analysis. There are several IFN-γ-induced proteins with antiviral activity, which can inhibit viral protein synthesis, edit viral sequences, degrade RNA, and impair the transport of viral nucleocapsid to the nucleus; this can, in turn, explain why higher IFN-γ levels are beneficial and related to protection in HESN individuals (36, 37). This is the first report describing high cytotoxic capacity as well as higher IFN-γ and MIP-1α production by NK cells in high-risk MSM, showing that these mechanisms are conserved among different cohorts of HESN individuals. However, a mature phenotype and higher frequency of CD57^+^/NKG2C^high^ NK cells are poorly reported features in HESN individuals. In fact, higher frequencies of fully mature and CD57^+^/NKG2C^+^ NK cells have only been reported in a cohort of serodiscordant couples from Brazil, with NK cells showing increased CD107a and IFN-γ expression (14). Thus, higher frequencies of CD57^+^/NKG2C^high^ NK cells in our MSM group as well, suggest that these cells could be involved in natural resistance to HIV infection.

It has been extensively reported that NK cells need priming to acquire functional competence, including cytokines, such as type I IFN, IL-12, IL-15, and IL-18 to acquire adequate cytotoxicity (38–40). Experimental models of persistent viral infections have shown that lower, but persistent, levels of cytokines induce NK cell activation for long periods (41, 42). The high-risk MSM have increased sexual exposure to different pathogens or antigens; this drives the environment required for the priming process, which explains the enhanced cytotoxicity and IFN-γ production that is characteristic of an experienced NK cell population.

Although proinflammatory stimulus may explain some long-term adaptations in conventional NK cells, the response of CD57^+^/NKG2C^high^ NK cells to a proinflammatory stimulus is low or absent due to decreased expression of cytokine receptors (43). However, some infections such as hantavirus, chikungunya, and HIV are effective at activating or expanding the compartment of CD57^+^/NKG2C^high^ NK cells generated after HCMV infection (28, 29, 44). Although the mechanism underlying this phenomenon is unclear, some of these responses have been attributed to elevated HLA-E expression (28). The upregulation of HLA-E, the ligand of NKG2C, is a common feature found in viral infections, such as dengue, hantavirus, and HIV (28, 45, 46). The engagement of NKG2C, the signature activating receptor of CD57^+^/NKG2C^high^ NK cells, leads to polyfunctional responses characterized by degranulation of cytolytic molecules as well as TNF-α and IFN-γ release (29). In this way, cross-linking of NKG2C alone is sufficient to drive IFN-γ production in CD57^+^/NKG2C^high^ NK cells (47), contradicting previously described requirements for NK cell activation (48).

When the frequency of STIs was evaluated according to the nature of the causative agent, high-risk MSM showed lower frequencies of viral STIs, but not bacterial ones. The reduced frequency of viral STIs in high-risk MSM could, potentially, be explained by this phenomenon, where CD57^+^/NKG2C^high^ NK cells, activated through NKG2C by HLA-E, induce a strong antiviral response mediated by CD57^+^/NKG2C^high^ NK cells that can protect these individuals against a variety of viral infections, including HIV.

In both cases, higher frequencies of experienced conventional NK cells and CD57^+^/NKG2C^high^ adaptive NK cells indicate that there is a well-trained and powerful army of effector cells that can mediate a rapid and strong response against a variety of pathogens. As observed in our cohort, where fully mature and CD57^+^/NKG2C^high^ adaptive NK cells might play an important role in natural resistance to HIV infection. Via either adaptive responses, generated after HIV contact or heterologous responses, generated by high exposure to other pathogens, NK cells might mediate an adequate control, avoiding the establishment of HIV infection.

In addition to the particular phenotypic and functional features, some genetic characteristics differed between groups. Protective alleles *HLA-B^∗^18* and *HLA-B^∗^39* were enriched in the high-risk MSM group. In babies born to seropositive mothers, the frequencies of the *HLA-B^∗^18* allele were associated with a significantly lower risk of early HIV-1 transmission via breastfeeding (49); likewise, *HLA-B^∗^18* has also been shown to be related to HIV protection in serodiscordant couples (50). The *HLA-B^∗^39* allele has been associated with HIV protection in some studies (51, 52), although less often.

Besides, the protective phenotype of *KIR3DL1/S1*, in combination with *HLA-Bw4* in the homozygous state, was found in 30.7% of high-risk MSM compared with a rate of 8% in low-risk MSM. HLA alleles containing the Bw4 epitope act as KIR ligands, while alleles with the Bw6 epitope do not. NK cells recognize self-HLA through KIR receptors, generating an inhibitory signal preventing auto aggression. Downregulation of HLA molecules at the cell membrane, a common phenomenon in viral infections, allows NK cell activation by “missing-self recognition” (53). *KIR3DL1/S1* + NK cells educated in the context of Bw4 alleles exhibit a stronger capacity to kill HIV-1-infected cells and, as a consequence, patients with *KIR3DL1/S1* + NK cells exhibit lower viral load outcomes during the infection (54–57). Boudreau et al. reported that *KIR3DL1* and *Bw4-80I* partnerships endow NK cells with the greatest reactivity against HLA-negative targets, whereas NK cells exhibiting *KIR3DL1* in combination with *HLA-Bw4*, not 80I, demonstrate intermediate responsiveness and *Bw4-/KIR3DL1* + NK cells are poorly responsive (55). This evidence suggests that the expression of HLA-Bw4 alleles, in combination with *KIR3DL1/S1*, can be associated with resistance to both progression to AIDS and HIV infection in different HESN cohorts (33, 58, 59).

The data presented in this article suggest that NK cells of high-risk MSM present functional characteristics that could be related to the fact that MSM remain seronegative despite years of high-risk practices. The higher frequency of CD57^+^/NKG2C^high^ NK cells exhibited by this population was correlated with these characteristics, indicating this subset’s important role during HIV exposure. Interestingly, similar anti-HCMV titers found between the two groups indicate that inducing CD57^+^/NKG2C^high^ NK cells can be modulated by several factors that increase their frequency and impact their functional capacity, opening up an interesting field of research in HIV treatment based on boosting NK cells.

## Data Availability Statement

All datasets generated for this study are included in the article/Supplementary Material.

## Ethics Statement

The studies involving human participants were reviewed and approved by the Ethics committee Universidad de Antioquia. The patients/participants provided their written informed consent to participate in this study.

## Author Contributions

LF-Á, JH, and WZ designed the research, analyzed the data, and wrote the manuscript. LF-Á, YB, KR, and AO-G performed the research. PV contributed to analytical tools. WZ supervised the project. All authors contributed to the article and approved the submitted version.

## Conflict of Interest

The authors declare that the research was conducted in the absence of any commercial or financial relationships that could be construed as a potential conflict of interest.
