# Supplementary material for: NK Cell Activity and CD57+/NKG2Chigh Phenotype Are Increased in Men Who Have Sex With Men at High Risk for HIV
Source: Front Immunol. 2020 Sep 11;11:537044. doi: 10.3389/fimmu.2020.537044 (PMC7517039; doi:10.3389/fimmu.2020.537044)

Supplementary Material

***Supplementary Table 1*.** Primer sequences and PCR conditions for gene expression evaluation.

| **Gene** | **Primer Sequence** | **T melting** |
| --- | --- | --- |
| **PGK** | Fw 5`-GTTGACCGAATCACCGACC-3`  Rv 5`-TCGACTCTCATAACGACCCGC-3` | 60°C |
| **RANTES** | Fw 5`-CCATGAAGGTCTCCGCGGCA-3`  Rv 5`-GTGGGCGGGCAATGTAGGCAA-3` | 64°C |
| **MIP -1β** | Fw 5`-CTGCCTTCTGCTCTCCAGCG-3`  Rv 5`-GGAGCAGAGGCTGCTGGTCT-3` | 60°C |
| **MIP-1α** | Fw 5`- TGCATCACTTGCTGCTGACACG-3`  Rv 5`- CAACCAGTCCATAGAAGAGG-3` | 61°C |
| **Perforin** | Fw 5`- CCGCTTCTACAGTTTCCATGT-3`  Rv 5`-GTGCCGTAGTTGGAGATAAGC-3` | 52°C |
| **Granzyme** | Fw 5`- CACTGTTGGGGAAGCTCCAT-3`  Rv 5`-TGGGGGATGGGTCTTTTCAC-3` | 54°C |
| **IFN-γ** | Fw 5`-TCGTTTTGGGTTCTCTTGGC -3`  Rv 5`-TCTGTCACTCTCCTCTTTCCAA-3` | 59°C |
| **IL-22** | Fw 5`-CCCTATATCACCAACCGCAC-3`  Rv 5`-CACTCATACTGACTCCGTGG-3` | 58°C |
| **TNF-α** | Fw5`- CCCATGTTGTAGCAAACCCTC-3`  Rv 5`- TATCTCTCAGCTCCACGCCA-3` | 60°C |

All amplifications were done with the same protocol. Initial enzyme activation step of 94°C for 10 min. Then, denaturation at 94°C for 10 sec; annealing at T° melting for 30 sec; extension at 72°C for 30 sec, 40 times, and a final extension step at 72°C for 2 min.


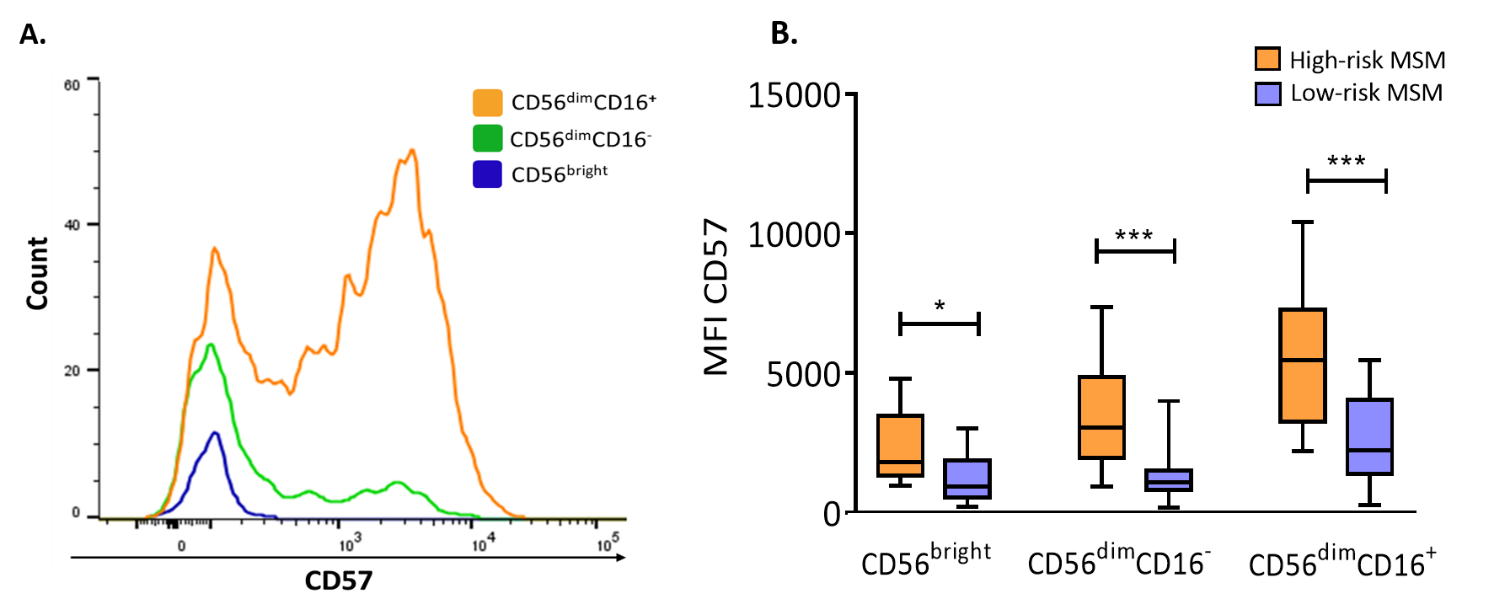


***Supplementary figure 1.* *NK cells from high-risk MSM exhibit a mature phenotype compared with those in low-risk MSM.* A.** Histogram representing the CD57 expression on NK cell subpopulations. CD56^bright^, the immature population, has little or no CD57 expression. CD56^dim^CD16^+^ cells show a higher frequency and intensity of this marker’s expression according to their maturation stage, while CD56^dim^CD16^−^ cells show intermediate expression **B.** The MFI of CD57 expression on NK cell subpopulations between the study groups. The line inside the box indicates the mean, and whiskers indicate min. to max. value. n: 15;12. Statistical evaluations were performed using Mann–Whitney U or unpaired *t*-test. *p<0.05, **p<0.01, and ***p<0.001.

***Supplementary figure 2.*** Correlation between frequency of memory NK cells and sexual partners in the last three months

***Supplementary figure 3.*** Correlation between frequency of memory NK cells and lifetime sexual partners without values over 2000 sexual partners

***Supplementary Table 2*.** HLA-B alleles distribution among groups


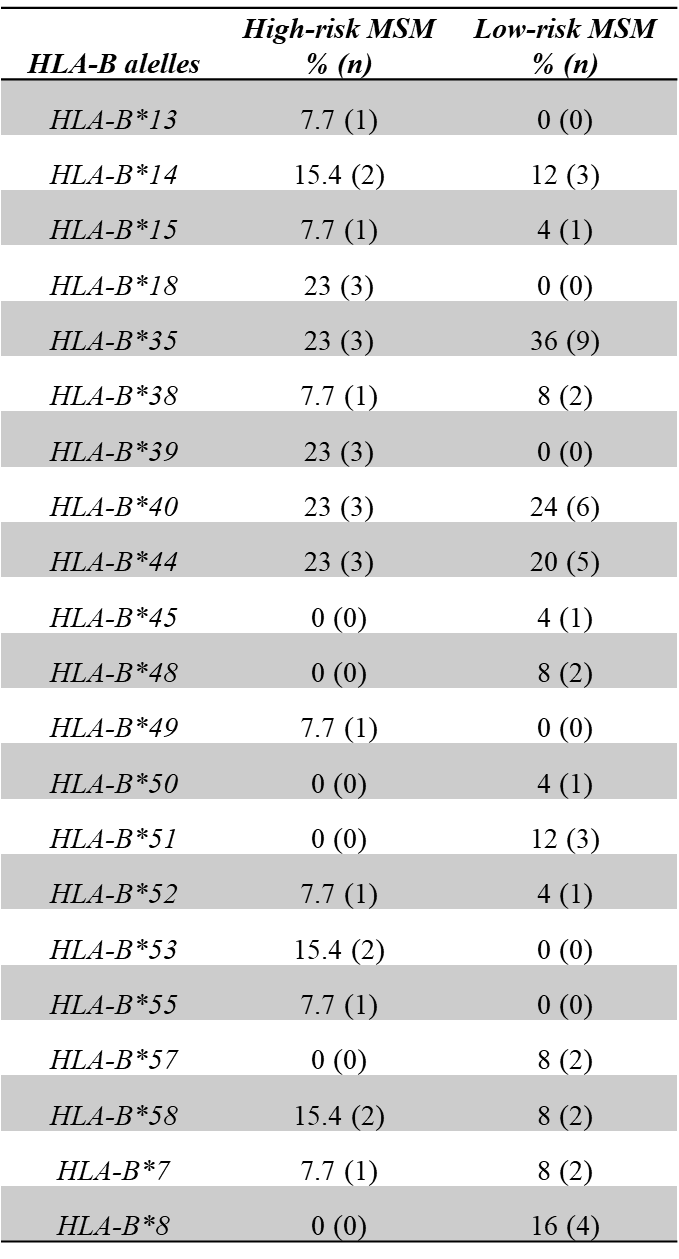


***Supplementary Table 3*.** KIR alleles distribution among groups


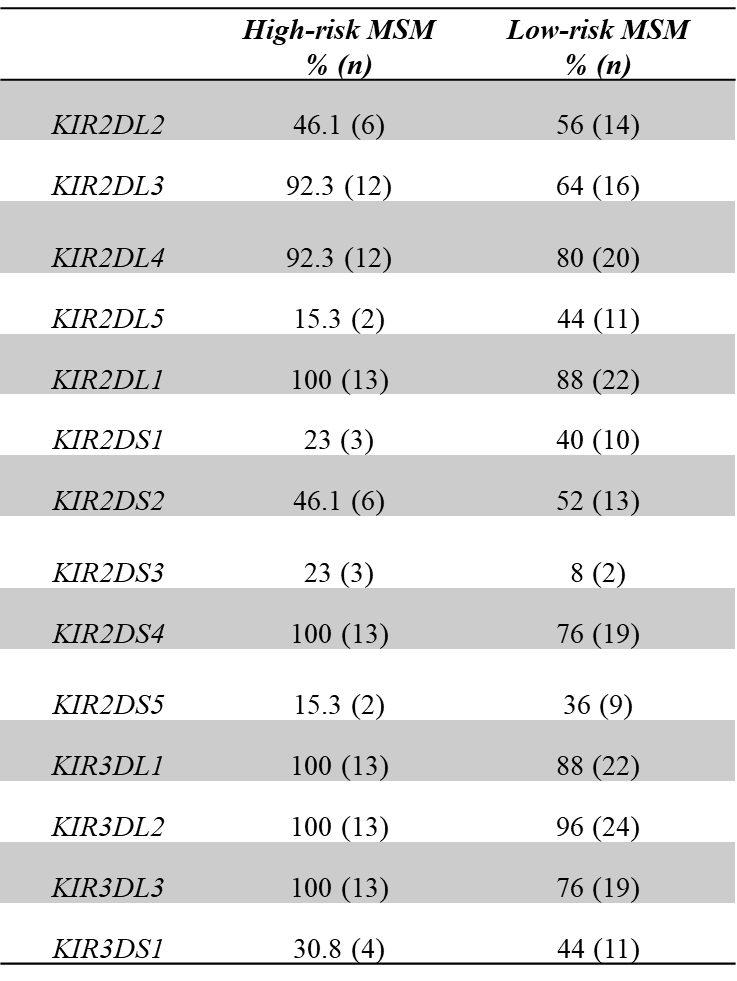

Supplement: Supplementary file 1 [file Table_1.DOCX]
